# Hyperspectral imaging of human liver allografts for prediction of initial graft function

**DOI:** 10.1007/s00423-024-03497-4

**Published:** 2024-10-14

**Authors:** Franziska Vogt, Tristan Wagner, Shadi Katou, Felicia Kneifel, Thomas Vogel, Haluk Morgül, Philipp Houben, Philip Wahl, Andreas Pascher, Sonia Radunz

**Affiliations:** 1 Department of General, Visceral and Transplant Surgery, University Hospital Münster, Münster, Germany; 2 Diaspective Vision GmbH, Strandstraße 15, 18233 Am Salzhaff, Germany; 3 Department of General, Visceral and Transplant Surgery, University Hospital Essen, Hufelandstr. 55, 45147 Essen, Germany

**Keywords:** Early allograft dysfunction, Human, Hyperspectral imaging, Liver transplantation, Normothermic machine perfusion

## Abstract

**Purpose:**

Ischemia reperfusion injury represents a significant yet difficult to assess risk factor for short- and long-term graft impairment in human liver transplantation (LT). As a non-invasive, non-ionizing tool, hyperspectral imaging (HSI) is capable of correlating optical properties with organ microperfusion. Hence, we here performed a study of human liver allografts assessed by HSI for microperfusion and prediction of initial graft function.

**Methods:**

Images of liver parenchyma of 37 human liver allografts were acquired at bench preparation, during normothermic machine perfusion (NMP), if applicable, and after reperfusion in the recipient. A specialized HSI acquisition software computed oxygen saturation (StO2), tissue hemoglobin indices (THI), near infrared perfusion indices (NIR), and tissue water indices (TWI). HSI parameters were analyzed for differences with regard to preservation technique, reperfusion sequence and presence of early allograft dysfunction (EAD).

**Results:**

Organ preservation was performed by means of NMP (*n* = 31) or static cold storage (SCS; *n* = 6). Patients’ demographics, donor characteristics, presence of EAD (NMP 36.7% vs. SCS 50%, *p* = 0.6582), and HSI parameters were comparable between both groups of preservation method. In organs developing EAD, NIR at 1, 2, and 4 h NMP and after reperfusion in the recipient was significantly lower (1 h NMP: 18.6 [8.6–27.6] vs. 28.3 [22.5–39.4], *p* = 0.0468; 2 h NMP: 19.4 [8.7–30.4] vs. 37.1 [27.5–44.6], *p* = 0.0011; 4 h NMP: 26.0 [6.8–37.1] vs. 40.3 [32.3–49.9], *p* = 0.0080; reperfusion: 13.0 [11.5–34.3] vs. 30.6 [19.3–44.0], *p* = 0.0212).

**Conclusion:**

HSI assessment of human liver allografts is feasible during organ preservation and in the recipient. NIR during NMP and after reperfusion might predict the onset of EAD. Larger trials are warranted for assessment of this novel technique in human LT.

## Introduction

For patients with end-stage liver disease, liver transplantation remains the only curative treatment option. Transplant specialists have been called upon to expand the traditional organ donor pool for several years now to offer liver allografts to as many candidates as possible on the liver transplant waiting lists, resulting in a considerable variability in early and late graft function.

The return of dynamic organ preservation methods, especially normothermic machine perfusion (NMP), enables the successful transplantation of extended criteria donor (ECD) organs that would have until recently been rejected [1–4]. Besides providing valuable information about donor organ viability as assessed through lactate clearance, perfusate pH, glucose metabolism, bile production and arterial blood flow [5], organ reconditioning might be achievable utilizing NMP [6].

The former reluctance to a regular acceptance of ECD grafts is typically based on an increased risk of primary non-function and early allograft dysfunction (EAD) [7]. EAD represents the clinical manifestation of severe ischemia-reperfusion injury due to a variety of donor, recipient, and perioperative factors, and has been shown to result in inferior long-term graft and patient survival [8, 9]. The prediction of EAD is either based on donor characteristics and histological assessment of zero-time biopsies, or liver function tests in the recipient in the initial postoperative course [10, 11]. So far, there is no universal method to predict EAD in liver transplantation timely and safely. Especially in the setting of static cold storage (SCS) organ preservation, when organ viability assessment is not possible, there is a considerable interest in non-invasive tools to monitor organ quality before transplantation.

In kidney transplantation, hyperspectral imaging (HSI) allograft assessment was capable of delivering quantitative information on organ viability and performance. On HSI assessment, transplant kidneys with delayed graft function (DGF) displayed significantly decreased allograft oxygenation and microperfusion after reperfusion in the recipient [12, 13]. A proof of concept pilot study was able to prove the technical feasibility of the combination of HSI and liver NMP [14].

In short, HSI allows for non-invasive quantification of perfusion and oxygenation of various organs in 2D using imaging measurements in a wavelength range between 500 and 1000 nm, without the application of radiation or contrast agents. The HSI system requires approximately 6 s for imaging, and using the analysis software TIVITA^®^ Suite Tissue (Diaspective Vision GmbH, Am Salzhaff, Germany) the obtained images are assessed with regard to superficial oxygen saturation (StO2), hemoglobin (THI), oxygen saturation at a depth of about 6 mm (NIR), and water concentration (TWI) [15]. Due to different spectral properties of different tissue contents, diagnostic information can thus be obtained about morphology, possible pathologies and indirectly about metabolic function. In a previous study, we were able to demonstrate that StO2 and THI obtained during NMP predicted perfusate lactate levels at the same time [16].

Hence, objective of this study was to evaluate the applicability of HSI in monitoring allograft microperfusion in the setting of human liver transplantation and to investigate differences in HSI parameters in organs with and without EAD. In addition, we examined whether the type of organ preservation (NMP or SCS) or reperfusion sequence (sequential or simultaneous) resulted in HSI-detectable differences in microperfusion.

## Materials and methods

### Study population

Adult patients who underwent liver transplantation at our center from March 2021 to October 2021 were included in the study. The study was conducted in accordance with both the Declarations of Helsinki and Istanbul, and approved by the local ethics review committee of Westfalen-Lippe, Münster, Germany (ID 2021-574-f-S). The requirement for written informed consent for the study was waived.

### Organ preservation

Following organ procurement, all liver allografts were preserved by static cold storage (SCS) using histidine-tryptophan-ketoglutarate solution (Custodiol^®^ HTK Solution) or University of Wisconsin solution (Belzer UW^®^ Cold Storage Solution) for organ preservation during transportation from the donor to the recipient hospital. Further organ preservation at the recipient hospital was performed by means of NMP or SCS. NMP using OrganOx^®^ metra was preferred whenever a perfusion time of at least 6 h could be accepted. No specific donor or recipient criteria were used as inclusion or exclusion criteria for the application of NMP.

### Hyperspectral imaging

The commercially available TIVITA hyperspectral camera (TIVITA^®^, Diaspective Vision GmbH, Germany) was used for hyperspectral imaging. Technical and physical background, the function of hyperspectral imaging as well as the algorithms of the different parameters were presented by Holmer et al. in a very detailed publication [15]. In brief, the technology is based on the fact that due to the inhomogeneity of biological tissue, light is multiply scattered and absorbed mainly by chromophores such as hemoglobin and water as it propagates through the tissue. The light reflected from the tissue, transmitted and detected by HSI, contains quantitative diagnostic information about the examined tissue [17].

In order to avoid reflections during imaging, HSI measurements were performed with the room light switched off. The camera was set at a distance of 50 cm to the liver allograft and a focal length 25 mm was utilized. Imaging measurements were performed at the following predefined time points: after bench preparation of the donor organ; in case of normothermic machine perfusion after 1, 2 and 4 h and at the end of machine perfusion; and after final reperfusion in the recipient. Subsequently, 10 markers of a size of 20 mm were placed on each image and the arithmetic mean values were formed to quantitatively assess the HSI parameters of each liver allograft.

### Liver transplantation

Orthotopic liver transplantation was performed using the modified piggyback technique according to Belghiti with an end-to-side cavo-caval anastomosis and temporary portocaval shunting [18]. Depending on recipient hemodynamic status during the anhepatic phase, allograft reperfusion was performed sequentially or simultaneously. The level of the arterial anastomosis was dependent on the size and quality of the recipient and donor arteries; usually, the gastroduodenal artery confluence was used. Postoperative immunosuppression consisted of tacrolimus 0.1 mg/kg adjusted to a trough level of 6–8 ng/mL, mycophenolate mofetil 1 g bid, and steroids, tapered and withdrawn within six weeks.

### Statistical analysis

The following recipient characteristics were assessed: age, sex, body mass index (BMI), liver disease, model of end-stage liver disease (MELD) score. The following donor variables were assessed: age, sex, height, weight, BMI, donor risk index (DRI). The following surgical data were collected: method of organ preservation, cold ischemia time (CIT), warm ischemia time (WIT), reperfusion sequence. The following outcome variables were recorded: presence of EAD, length of hospital stay, patient and graft survival. EAD was defined as the presence of one or more of the following: bilirubin ≥ 10 mg/dL on day 7, INR ≥ 1.6 on day 7 or ALAT or ASAT > 2000 IU/L within the first 7 days [9].

Data collection and statistical analysis were performed using Microsoft Excel 2010 (Microsoft Corporation, Redmond, WA, USA) and GraphPad Prism 10 for macOS version 10.0.2 (GraphPad Software, San Diego, CA, USA). Logistic regression analysis was performed using IBM SPSS Statistics (version 23.0 for Windows, SPSS, Inc., Chicago, IL, USA). All data were tested for normality using the D’Agostino&Pearson omnibus normality test. Categorical variables are presented as percentages and continuous variables as median [interquartile range], unless stated otherwise. Differences between groups were tested using Fisher’s exact test or chi-square test for categorical variables, and unpaired t test or Mann-Whitney test for continuous variables, as appropriate. Graft and patient survival were evaluated using the Kaplan-Meier method and compared with the log-rank test. The reference point for all calculations of survival was the day of liver transplantation. A *p* value ≤ 0.05 (two-tailed) was considered to be significant.

## Results

### Study population

The study population comprises 37 human liver allografts transplanted at our center between February and November 2021. All donor organs were derived from donation after brain death. Six liver allografts were preserved by SCS for the entire preservation period while 31 liver allografts were preserved by post-SCS NMP. Duration of NMP was 15.6 [11.5–18.4] hrs.

The recipient and donor characteristics as well as surgical details of both preservation groups are shown in Table 1. There were no differences with regards to recipient age, sex and BMI or underlying liver disease. Donor characteristics as represented by DRI were comparable between the groups. CIT was significantly shorter in the NMP group (7.2 [5.6–7.9] vs. 9.1 [6.6–12.7] hrs, *p* = 0.0091).

**Table 1:** Patients’ demographics, donor characteristics and surgical details with regards to organ preservation method

	NMP (*n* = 31)	SCS (*n* = 6)	*p*
Age (years)	54 [40–62]	57 [51–68]	0.3613
Male sex (%)	54.8	83.3	0.3682
BMI (kg/m2)	26.4 [23.7–31.1]	23.4 [22.1–33.3]	0.5118
MELD score	21 [9–32]	17 [10–40]	0.6357
Indication for LT			0.6793
Alcoholic cirrhosis (%)	12.9	0	
Hepatitis B (%)	9.7	0	
Hepatitis C (%)	0	0	
HCC (%)	22.6	33.3	
NASH (%)	6.4	0	
Other (%)	48.4	66.7	
DRI	1.560 [1.366–1.779]	1.656 [1.349–1.961]	0.4452
CIT (h)	7.2 [5.6–7.9]	9.1 [6.6–12.7]	**0.0091**
NMP duration (h)	15.6 [11.5–18.4]	n.a.	
WIT (min)	40 [35–46]	50 [43–61]	0.1864

normothermic machine perfusion, NMP; static cold storage, SCS; hepatocellular carcinoma, HCC; non-alcoholic steatohepatits, NASH; donor risk index, DRI; cold ischemic time, CIT; warm ischemic time, WIT

Reperfusion was performed simultaneously in 18 patients and sequentially, i.e. as primary portal vein reperfusion, in 19 patients. WIT was significantly longer in the simultaneous reperfusion group (46 [41–60] vs. 39 [30–45] min, *p* = 0.0025).

### Early allograft dysfunction

**Fig. 1:**
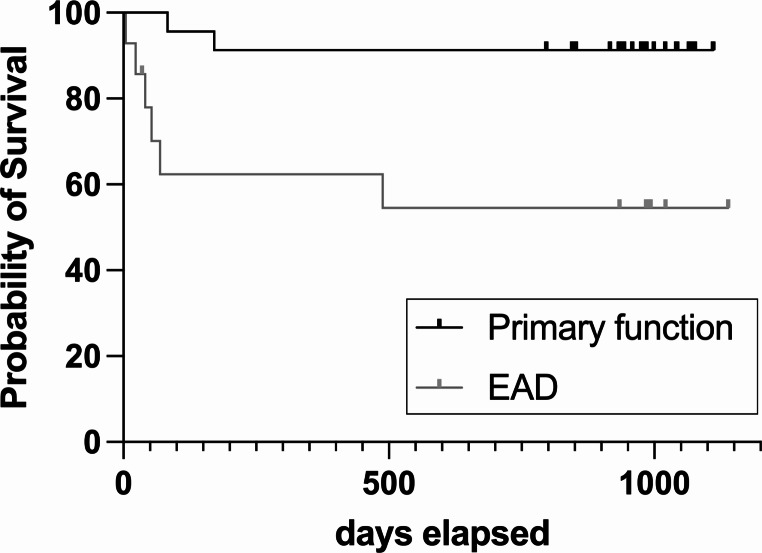
Patient survival according to initial graft function (*p* = 0.0062)

EAD was detected in 50.0% of patients in the SCS group and in 35.5% of patients in the NMP group (*p* = 0.6534). EAD was diagnosed due to elevated ASAT levels in all three patients in the SCS group. In the NMP group, seven patients met the criteria due to elevated ASAT levels, three patients demonstrated elevated bilirubin levels and in one patient both were present. In simultaneous reperfusion, EAD was present in 38.9% of patients, while in sequential reperfusion, EAD was detected in 36.8% (*p* > 0.9999). Recipients with a primarily functioning allograft and recipients with EAD did not differ with regards to age (53 [47–65] vs. 59 [41–64] yrs, *p* = 0.9806), predominance of male sex (56.5% vs. 64.3%, *p* = 0.7379), or BMI (26.3 [23.1–30.4] vs. 26.2 [23.1–37.0] kg/m2, *p* = 0.5217). Preoperative recipient liver function as assessed by MELD score was significantly worse in recipients with EAD (31 [16–40] vs. 15 [8-25], *p* = 0.0241). CIT (primary function 7.4 [5.8–8.7] vs. EAD 7.2 [6.0-8.2] hrs, *p* = 0.7426) as well as WIT (primary function 39 [35–50] vs. EAD 45 [40–62] min, *p* = 0.1330) were comparable between groups. Length of hospital stay did not differ significantly between groups according to initial graft function (primary function 16 [11-33] vs. EAD 31 [17–45] days, *p* = 0.1358). Graft loss was significantly more frequent in recipients with EAD (42.9% vs. 8.7%, *p* = 0.0070), and patient survival was significantly worse among recipients with EAD (Fig. 1, *p* = 0.0062). Two patients had to undergo early re-transplant on postoperative day (POD) 5 and day 18, respectively, due to severe graft dysfunction. In four patients, graft failure resulted in patient death on POD 4, POD 23, POD 53, and POD 69, respectively. In patients with a primarily functioning graft, two patients died with a functioning graft due to invasive tuberculosis within three months post-transplant and due to recurrent HCC within six months post-transplant, respectively.

### HSI assessment

**Fig. 2:**
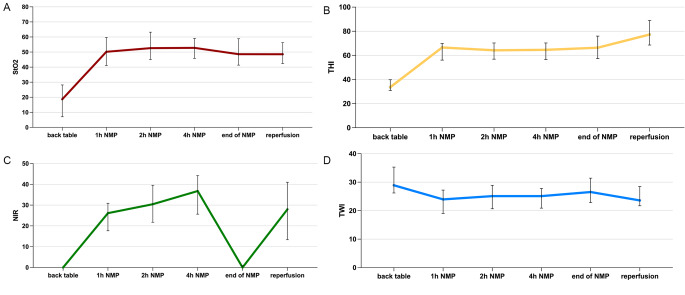
HSI parameters at bench preparation, during normothermic machine perfusion (NMP) and after reperfusion (**A**: tissue oxygenation (StO2), **B**: tissue hemoglobin index (THI), **C**: near-infrared perfusion index (NIR), and **D**: tissue water index (TWI))

HSI assessment of human liver allografts was feasible at bench preparation, during NMP and after reperfusion in the recipient. The course of the measured HSI parameters StO2, NIR, THI, and TWI at bench preparation, during NMP, and after reperfusion in the recipient are given in Fig. 2. Naturally, all perfusion parameters improved when compared between the unperfused status at bench preparation and the perfused status during NMP. This significant improvement was reached within one hour of NMP for all parameters. StO2, THI and TWI demonstrated stable values during the further course of NMP. NIR exhibited an additional improvement throughout the further course of NMP; however this did not reach statistical significance.

Different methods of organ preservation or different types of reperfusion sequence did not result in significant differences in HSI parameters after reperfusion in the recipient. Baseline values and measurements obtained after reperfusion in the recipient were comparable when stratified according to preservation method (Table 2). Merely, liver allografts preserved by SCS demonstrated lower NIR values after reperfusion in the recipient (13.2 [10.8–23.2] vs. 28.9 [16.8–42.3], *p* = 0.0601); however, this did not reach statistical significance.

**Table 2:** HSI parameters with regards to preservation method

	NMP (*n* = 31)	SCS (*n* = 6)	*p*
StO2 at backtable	20.3 [7.2–32.3]	13.7 [2.3–19.9]	0.1283
StO2 after reperfusion	48.6 [42.7–57.0]	48.5 [40.0-53.7]	0.8032
THI at backtable	33.9 [30.5–40.1]	33.1 [31.3–38.9]	0.7222
THI after reperfusion	77.3 [69.4–90.8]	76.0 [42.2–87.2]	0.1120
NIR at backtable	0.0 [0.0–0.0]	0.0 [0.0–0.0]	> 0.9999
NIR after reperfusion	28.9 [16.9–42.3]	13.2 [10.8–23.2]	0.0601
TWI at backtable	28.7 [26.1–35.4]	31.1 [25.2–35.1]	0.8885
TWI after reperfusion	23.5 [21.7–30.1]	26.2 [21.0-27.1]	0.7801

normothermic machine perfusion, NMP; static cold storage, SCS; tissue oxygenation, StO2; tissue hemoglobin index, THI; near-infrared perfusion index, NIR; tissue water index, TWI

**Fig. 3:**
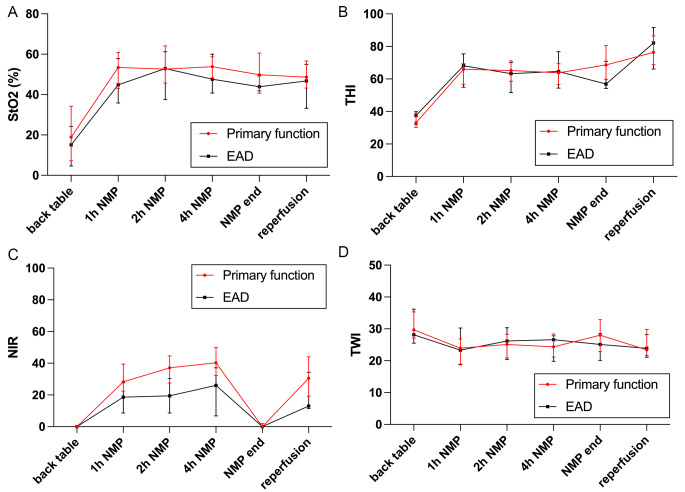
HSI parameters with regards to initial graft function (**A**: tissue oxygenation (StO2), **B**: tissue hemoglobin index (THI), **C**: near-infrared perfusion index (NIR), and **D**: tissue water index (TWI))

In organs with EAD, baseline values of HSI parameters at bench preparation were comparable to those of allografts with primary function. At 1, 2, and 4 h NMP and after reperfusion in the recipient, NIR was significantly lower in organs with EAD (1 h NMP: 18.6 [8.6–27.6] vs. 28.3 [22.5–39.4], *p* = 0.0468; 2 h NMP: 19.4 [8.7–30.4] vs. 37.1 [27.5–44.6], *p* = 0.0011; 4 h NMP: 26.0 [6.8–37.1] vs. 40.3 [32.3–49.9], *p* = 0.0080; reperfusion: 13.0 [11.5–34.3] vs. 30.6 [19.3–44.0], *p* = 0.0212). For StO2, THI, and TWI, this difference was not noticeable (Fig. 3).

## Discussion

Current challenges in liver transplantation that continue to arise include a persistent shortage of donor organs and limited donor quality [19]. To meet the ongoing demand, transplant specialists have come to continuously reevaluate donor organs formerly considered of too high a risk for allograft dysfunction, i.e. ECD organs. The use of liver transplants from older donors has increased, as has - due to the obesity epidemic - the incidence of steatotic transplants [7].

The re-establishment of machine perfusion for organ preservation supports the growing utilization of previously deemed non-transplantable allografts. Especially in ECD organs, the applicability of SCS is limited due to an increased susceptibility for ischemia-reperfusion injury (IRI). IRI leading to the formation of reactive nitrosaminic and oxidative species, loss of vascular regulation, impaired endothelial cell function and tissue perfusion, and finally tissue hypoxia may increase the risk of EAD [20, 21]. While preserved by NMP, allografts may be assessed for viability and may be recruited for transplantation despite initial refusal for transplantation [2, 22]. Indeed, several promising studies suggest that NMP not only allows functional testing of the donor liver, but also improves organ quality, which has led to a reduced number of rejected or discarded donor livers [1–6, 22–24]. Prolonged CIT on the other hand is an independent risk factor for impaired graft function, as well as for shortened patient survival, and even more so when advanced donor age and/or donor liver steatosis are present as well [25].

Currently, EAD constitutes the best-validated and clinically relevant parameter for early liver allograft assessment after liver transplantation [9]. As demonstrated in our study, recipients with EAD have an increased risk for graft loss and a significant worse patient survival. According to European Liver Transplant Registry (ELTR) data, the critical period for outcome after liver transplantation is the first year and primary non-function/dysfunction are the main causes for retransplantation [26]. Liver allografts with an initial poor function or even non-function still present a very considerable morbidity and mortality risk for recipients. Patients at risk for EAD need to be identified as early as possible for prompt targeted and supportive treatment or timely retransplantation. Hence, objective immediate assessment of allografts rather than subjective evaluation by an experienced transplant surgeon is still sought after.

Until today, the gold standard for objective evaluation of liver allografts remains biopsy of the liver, which is usually the basis for acceptance or rejection especially of ECD organs. However, it is critical to note that awaiting histology results may prolong CIT and that biopsies might be subject to sampling and observer bias [25]. Benkö et al. demonstrated that non-invasive assessment of liver microcirculation and hemoglobin oxygenation using Oxygen-to-See (O2C) spectrometry yielded predictive factors for EAD following liver transplantation [27]. Adequate microperfusion sufficiently supplies oxygen and nutrients to the liver and clears metabolic waste products; changes in microperfusion result in reduced oxygen availability and impaired nutritive blood flow.

Hence, camera-based real-time techniques such as HSI could be of great interest in liver allograft viability assessment. HSI is a non-invasive, non-contrast, safe, and rapid method of assessing, among other parameters, the hepatic microcirculation in vivo. Its medical fields of application are diverse: HSI has been shown to determine resection margins and optimal anastomotic regions in, for example, colorectal surgery [28–30], to quantify hepatic steatosis [31], to detect, characterize and classify tumors [17], and to predict delayed graft function (DGF) in kidney transplantation [12, 13].

Aim of the current study was to analyze the applicability of HSI as a tool for predicting liver transplant outcome, preferably during organ preservation or at least upon reperfusion in the recipient. In our study, HSI provided valuable information about the microperfusion of liver allografts during NMP as well as in the recipient. Decreased NIR levels during NMP and after reperfusion in the recipient were significantly associated with an increased risk of EAD occurrence. Two findings emerge from this: first, the application of HSI in the setting of liver transplantation is feasible and safe, regardless of the organ preservation method; and second, it is reasonable to assume that decreased NIR levels are associated with impaired organ microperfusion, which is accompanied by an increased risk of EAD.

We applied the Olthoff criteria for EAD as these variables are easy to identify, objective, and widely in use [9]. Since their first description, and even before, many attempts have been made to define EAD and to predict short- and long-term survival of transplant allografts and recipients based on different clinical parameters in order to take preventive action, if necessary. In recent years, several studies have been published criticizing that the “classical” EAD criteria are too imprecise or that there are better scores to predict graft loss [10, 32–34]. Novel risk scores such as the L-GrAFT score are reported to outperform long-standing scores, i.e. binary EAD or MEAF score [10, 35]. Nevertheless, calculation of the L-GrAFT score is rather complex: for calculation of the L-GrAFT_7_ score, 28 data entries are required, a software or an online tool is not yet available, and its logarithmic transformation hampers daily use. An actual advantage in the clinical setting would be a truly reliable score based on pre-transplant parameters for up-front decision making during organ allocation. Furthermore, future scores should implement the application of machine perfusion or its parameters as additional variables. In real-life settings, ease of use will always win out over improved sensitivity and specificity.

There are some limitations to the applicability of HSI as well: imaging requires a relatively close, direct view of the organ, i.e. transcutaneous measurements are not possible. In addition, placing of the region of interest (ROI) is user-dependent and might carriy a risk of information bias. So far, microcirculatory assessment tools have not made it to clinical practice, despite promising results in research studies. Ease of use remains a key element, when implementing new tools in surgical settings, especially when interrupting well-trained, smooth and quick moves of a specialized surgeon. In today’s times of further worsening donor organ quality, the application may be of greater consequence than at the time of earlier developments such as O2C.

Our study itself has also some limitations: the study cohort was limited to 37 liver allografts, without randomization, and performed at a single center. Nevertheless, this study is the largest to date in the field HSI application in liver transplantation. Due to the relatively high rate of EAD, regardless of the organ preservation method, differences in microperfusion depending on the initial graft function could be observed. As these differences in HSI values are not evident during bench preparation, HSI assessment of the liver may not support the decision to accept or discard an organ prior to cell-based reperfusion, i.e. during NMP or in the recipient, which could save valuable resources. For now, subjective visual assessment of the organ by the procurement surgeon and, if necessary, liver biopsy will remain the methods of choice. Considering that cell-based perfused livers displayed differences between NIR values as shown, HSI could possibly be applied during procurement surgery prior to circulatory arrest and already provide valuable information at this point; however, further studies would be required to verify this hypothesis. In the future, routine measurements of hepatic microperfusion might contribute to the prediction of EAD in patients undergoing liver transplantation.

## Conclusion

Our study demonstrates that HSI assessment might serve as a reliable, simple and rapid tool for real-time monitoring of human liver allografts during organ preservation and after reperfusion in the recipient. HSI provides relevant information on hepatic microperfusion, which in turn is an indicator of IRI and correlates with initial graft function. During NMP, HSI might serve as a non-invasive viability assessment for timely detection of parenchymal damage resulting in allograft dysfunction. Further investigations are required to determine the predictive value of HSI regarding EAD.

## Data Availability

The data that support the findings of this study are available from the corresponding author upon reasonable request.
